# Backbone ^1^H, ^13^C, and ^15^N resonance assignments of the ligand binding domain of the human wildtype glucocorticoid receptor and the F602S mutant variant

**DOI:** 10.1007/s12104-018-9820-9

**Published:** 2018-04-17

**Authors:** Christian Köhler, Göran Carlström, Stefan Tångefjord, Tineke Papavoine, Matti Lepistö, Karl Edman, Mikael Akke

**Affiliations:** 1 Respiratory, Inflammation and Autoimmunity, IMED Biotech Unit, AstraZeneca, Gothenburg, Sweden; 2 Discovery Sciences, IMED Biotech Unit, AstraZeneca, Gothenburg, Sweden; 3 Centre for Analysis and Synthesis, Department of Chemistry, Lund University, Lund, Sweden; 4 Biophysical Chemistry, Center for Molecular Protein Science, Department of Chemistry, Lund University, Lund, Sweden

**Keywords:** Glucocorticoid receptor, Nuclear receptors, Allostery, Ligand binding

## Abstract

The glucocorticoid receptor (GR) is a nuclear hormone receptor that regulates key genes controlling development, metabolism, and the immune response. GR agonists are efficacious for treatment of inflammatory, allergic, and immunological disorders. Steroid hormone binding to the ligand-binding domain (LBD) of GR is known to change the structural and dynamical properties of the receptor, which in turn control its interactions with DNA and various co-regulators and drive the pharmacological response. Previous biophysical studies of the GR LBD have required the use of mutant forms to overcome issues with limited protein stability and high aggregation propensity. However, these mutant variants are known to also influence the functional response of the receptor. Here we report a successful protocol for protein expression, purification, and NMR characterization of the wildtype human GR LBD. We achieved chemical shift assignments for 90% of the LBD backbone resonances, with 216 out of 240 non-proline residues assigned in the ^1^H–^15^N TROSY spectrum. These advancements form the basis for future investigations of allosteric effects in GR signaling.

**Electronic supplementary material:**

The online version of this article (10.1007/s12104-018-9820-9) contains supplementary material, which is available to authorized users.

## Biological context

The ability to regulate the expression of genes is an essential feature of higher organisms. Gene regulation responds to environmental changes, and is the basis for cell differentiation and morphogenesis. The glucocorticoid receptor (GR) is a ligand-activated transcription factor. It regulates genes that are linked to development, metabolism, inflammation, and the immune and stress responses (Beato 1989). GR comprises three domains: the disordered N-terminal domain (NTD), the DNA binding domain (DBD) and the ligand-binding domain (LBD). The hormone-free (apo) GR predominately resides in the cytoplasm, where it is both stabilized and kept in a ligand receptive state by various chaperones, including HSP40, HSP70 and HSP90 (Vandevyver et al. 2012). GR activation as a result of ligand binding leads to partial dissociation of the chaperone proteins followed by translocation into the nucleus (Vandevyver et al. 2012). Subsequently, GR will bind to specific genomic DNA sequences, where the receptor will recruit co-regulators and other transcription factors resulting in a context-dependent protein complex that will ultimately activate or repress gene transcription. In addition, there is also a fast non-genomic hormone response, which does not regulate gene transcription, but triggers fast signaling events in the cytoplasm (Kadmiel and Cidlowski 2013). Examples of non-genomic GR responses are the reduction of bronchial vascular blood flow or inhibition of specific kinases that are crucial for T-cell activation (Alangari 2010). Taken together, these processes result in a complex regulatory network that is highly dependent on partner protein expression patterns across different cells, combined with the prevalence of various receptor isoforms (Bledsoe et al. 2002; Kadmiel and Cidlowski 2013).

The LBD adopts a 3-layered globular fold, comprising 12 α-helices with a fully enclosed ligand binding pocket (Bledsoe et al. 2002). Ligand binding drives allosteric rearrangements of key areas on the domain surface (McInerney et al. 1998; Edman et al. 2015) including the dimerization interface and the activation function 2 (AF2). AF2 is located at the intersection of helices 3, 4 and 12 and binds to the canonical LxxLL motif of co-regulator proteins and as such has a key role in GR as a scaffolding protein (McInerney et al. 1998).

Molecular communication within the LBD and in between the GR domains forms the basis for its biological signaling. Allosteric modulation enables binding effects at one site to be transmitted to distal functional sites. The structural relationship between discrete sites is often inferred from structural comparisons of complexes with different effectors bound (Bledsoe et al. 2002; Edman et al. 2014). However, this approach lacks detail on how the allosteric signal propagates through the protein. To reach an in-depth molecular understanding of down-stream effects of GR allostery and its consequences for specific cell responses, it is critical to map the network of dynamical changes within the LBD that are triggered by binding different hormone ligands and co-regulators. Increased knowledge in this area may advance drug design to the stage where specific chemical motifs can be exploited to generate distinct structural states with cell and tissue specific pharmacology, giving the potential to reduce the adverse effect profile of the currently available drugs.

Biophysical characterization of the GR LBD has to date been hampered by its low stability and high aggregation propensity. To overcome these issues several stabilizing mutations have been described in the literature, but all involve the risk of affecting GR’s functional properties. One of the best characterized mutants is F602S (Ricketson et al. 2007), widely considered as the minimum mutation required to generate a sufficiently stable system for biophysical studies. However, the non-conservative F602S mutation is not only stabilizing, but also has agonistic effects (Ricketson et al. 2007), indicating that this and other mutant variants might not serve as optimal models of GR LBD signaling.

## Methods and experiments

### Protein expression and purification

Based on the conserved regions of the amino-acid sequences among GR orthologs, we estimated the domain boundaries of the DBD, hinge-region, and LBD (Fig. S1A), resulting in the design of four LBD constructs of different lengths: I500–K777, N514–K777, V521–K777 and T529–K777. All wildtype (wt) constructs were PCR amplified and cloned into the pET24a vector (Novagen) featuring an N-terminal His_6_-tag and a TEV protease cleavage site. The expression vector was transformed into Escherichia coli BL21(DE3) STAR, followed by high cell-density expression according to published protocols (Sivashanmugam et al. 2009). Cells were grown in TB medium to an OD of 5, followed by transfer into standard M9 minimal medium in D_2_O (cell medium 1) with increased amounts of ^13^C glucose (5 g/l) and ^15^NH_4_Cl (1 g/l) to account for the higher cell density. After exchange of the medium, cells were grown for another hour at 37 °C to allow for the discharge of unlabeled metabolites. The culture was subsequently cooled down and 100 µM dexamethasone and 1 mM isopropyl β-d-1-thiogalactopyranoside (IPTG) were added. The cells were further grown for 36 h at 16 °C. At the time of harvest a cell density of about 10 had been reached. This protocol led to an average, albeit uneven, deuteration level of approximately 70%. In order to produce perdeuterated protein samples, the same protocol was followed, except that deuterated glucose (^13^C^2^H glucose) was used (cell medium 2).

Expression of ^13^CH_3_-methionine labeled protein was achieved in a PASM-5052 autoinduction medium (cell medium 3). The aforementioned cell medium 1 was prepared without isotope enrichment and with the MgSO_4_ concentration increased to 2 mM. In addition, glucose was substituted with 0.5% glycerol, 0.05% glucose and 0.2% lactose. The medium was supplemented by 100 mg of all standard amino acids, except cysteine and tyrosine, which are synthesized by the cell from serine and phenylalanine, respectively. Further, 100 mg/l ^13^CH_3_-labeled methionine (Sigma) was added to the cell medium. Cells were grown to an OD of 0.6. After cooling down the culture to 16 °C, 100 µM dexamethasone was added and protein expression continued for 60 h. At the time of harvest, the cell density had reached approximately OD 15.

All buffers were degassed and contained 2 mM TCEP and 50 µM dexamethasone. The harvested cells were resuspended in lysis buffer (50 mM Tris pH 8, 10% glycerol, 1% CHAPS) supplemented by protease inhibitors (Complete, Roche) and DNAse. Cells were lysed by sonication. The cleared lysate was applied to a nickel affinity column equilibrated with wash buffer (50 mM Tris pH 8, 10% glycerol, 1% CHAPS, 60 mM NaCl) and eluted by a 300 mM imidazole gradient (Fig. S1B). His-tagged GR LBD was cleaved with TEV protease at 4 °C overnight while dialyzing against 50 mM Tris buffer pH 9. The cleaved protein was separated from the TEV protease, His-tag, and non-cleaved GR in a second nickel affinity step, using 50 mM Tris buffer pH 9 as running buffer (Fig. S1C). The purified protein contained small amounts of higher aggregates (Fig. S1C), which precipitated during sample concentration and did not affect the NMR spectra. The protein was stored at − 80 °C in 50 mM Tris buffer pH 9.

### NMR sample preparation

NMR samples were prepared by transferring the protein at low concentration (< 0.03 mM) to a 20 mM PO_4_ buffer, pH 7.5 with 10% (v/v) D_2_O, 1% CHAPS, 2 mM DTT, 50 µM dexamethasone, 0.02% NaN_3,_ and protease inhibitor, using a PD10 desalting column (GE healthcare). After adding a suitable co-regulator peptide, a fragment of the nuclear receptor coactivator 2 (KENALLRYLLDKDD), the sample was concentrated to 0.3 mM.

### NMR experiments

Backbone resonance assignments of wt GR LBD and the F602S mutant variant were carried out using ^1^H–^15^N TROSY (Pervushin et al. 1998) and TROSY-based 3D HNCA, HN(CO)CA and HNCO triple resonance experiments (Loria et al. 1999; Cavanagh et al. 2007). All triple resonance spectra were recorded at 25 °C on a perdeuterated ^2^H/^13^C/^15^N-labeled sample using a Bruker Avance 600 MHz spectrometer equipped with a TCI cryoprobe. To achieve good signal-to-noise, TROSY-based HNCA and HNCO spectra were recorded with 128 transients and 50 increments in each of the indirect dimensions, resulting in a total experiment time of about 6 days per spectrum. HN(CO)CA spectra could also be acquired with reasonable sensitivity, while experiments involving CB correlations were generally unsuccessful due to excessive line broadening. In addition a ^15^N-edited NOESY spectrum was acquired with a mixing time of 150 ms, 96 transients, and 80 and 60 increments in the indirect ^1^H and ^15^N dimensions, respectively, using a Bruker Avance III 800 MHz spectrometer equipped with a cryoprobe. ^1^H chemical shifts were referenced relative to the internal TSP signal, whereas ^15^N and ^13^C chemical shifts were referenced indirectly using the gyromagnetic ratios. NMR data were processed using the Bruker TopSpin software version 2.6 and analyzed using Sparky (Goddard and Kneller 2002).

## Assignments and data deposition

**Fig. 1:**
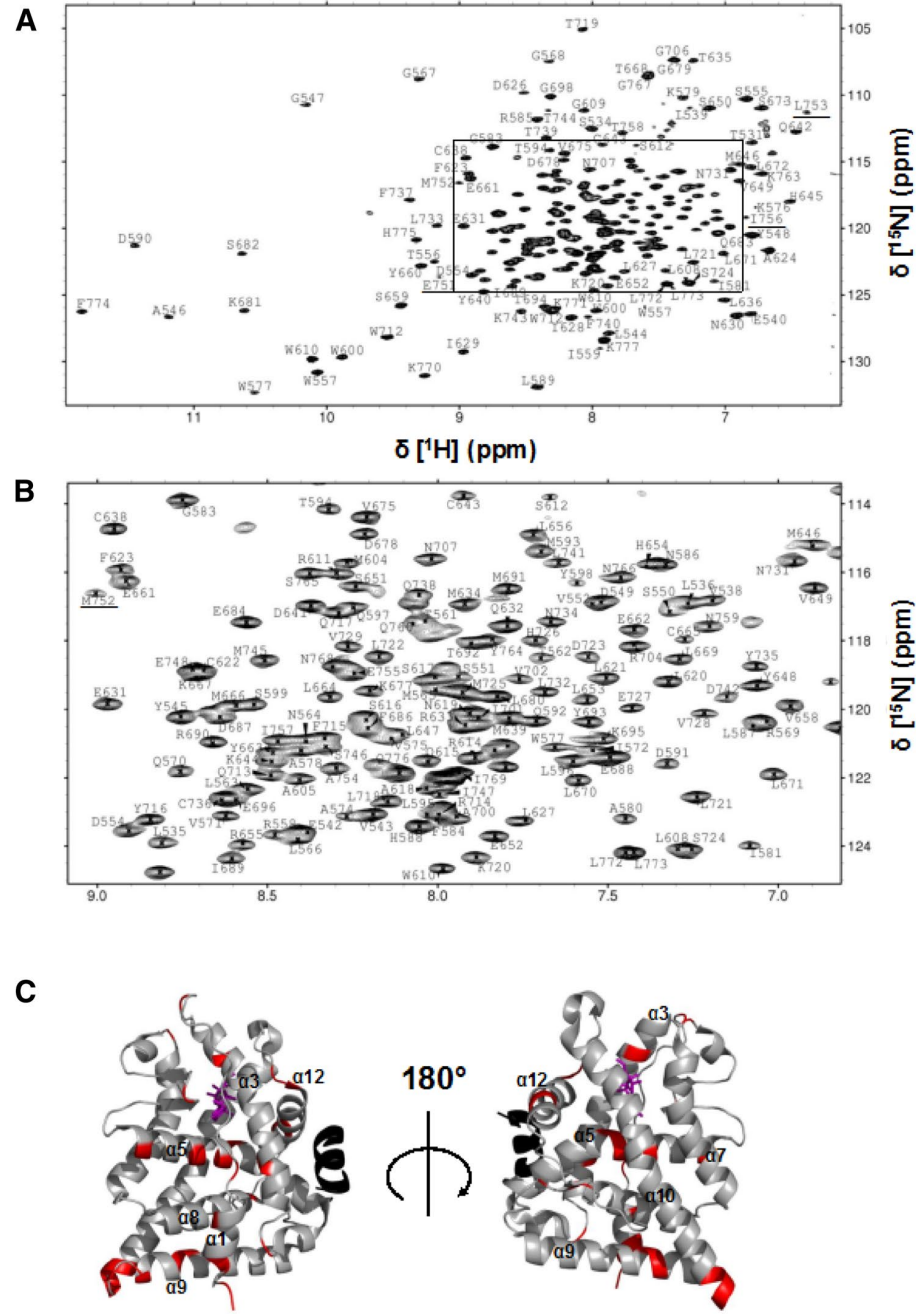
Assigned backbone amide resonances of wildtype GR LBD. **a** The assigned ^1^H–^15^N TROSY spectrum of wildtype GR LBD T529–K777. **b** Close-up view of the central region of the spectrum (boxed in **a**). Backbone resonances of residues in helix 12 are underlined; the weak intensities of these resonances are indicative of conformational exchange. **c** Non-assigned residues (red) mapped onto the X-ray structure of GR LBD (PDB id 4UDC) (Edman et al. 2015). Dexamethasone is highlighted purple. The co-regulator peptide is highlighted in black

Our efforts to establish a protocol for expression and purification of wt LBD reconfirm its low stability and high aggregation propensity, also apparent from its low expression yield and high prevalence in inclusion bodies. The ligand-free wt LBD was too unstable to be expressed in reasonable amounts and further aggregated even at low concentration. Initial trials to concentrate holo-wt LBD revealed the highest stability at pH 9. At pH values in the range beneficial for NMR spectroscopy (pH 5–7), the aggregation propensity is drastically higher, but can be counteracted by adding a suitable co-regulator peptide and a mild detergent such as CHAPS. To guide optimization of the LBD construct length we acquired ^1^H–^15^N TROSY spectra for a series of wt LBD constructs of different lengths (Fig. S2). The longer constructs contained disordered segments, which were successfully removed by stepwise N-terminal truncation, without any negative effects on the structural integrity of the LBD core, as evidenced by the correspondence of the backbone amide NMR-signals from the shortest LBD construct (T529–K777) and all longer fragments (Fig. S2). The LBD T529–K777 construct was used in all subsequent studies. In conclusion, the well resolved ^1^H–^15^N TROSY NMR spectrum indicates a folded protein suitable for chemical shift assignment and further biophysical studies (Fig. 1).

We assigned the backbone ^1^H, ^13^C, and ^15^N resonances using a combined strategy of sequential residue correlations based on HNCA, HNCO, and HN(CO)CA triple resonance experiments, and through-space NOE connectivities based on ^15^N-edited 3D NOESY experiments. The lack of CB correlations prompted us to employ isotopic unlabeling of all residue types, except Gly and Pro, as a means to obtain residue-specific resonance assignments. We followed a published protocol (Krishnarjuna et al. 2011) in which cells are grown in the aforementioned partly deuterated cell medium 1, supplemented by 100 mg/l of a given non-labeled amino-acid type. By comparing the ^1^H–^15^N TROSY spectra of the resulting protein samples with that of the fully labeled one, we could identify what residue type a given cross peak originates from; the reverse labeling of glycine residues was omitted since these residues can generally be identified from their characteristic amide resonances in the ^15^N up-field spectral region. Isotopic scrambling by the host cell metabolism caused ambiguous results for a few residue types, namely Arg, Lys, His, Met, and Asn. However, since residue interconversions are limited to chemically similar residue types, the unlabeling strategy still resulted in a reduction of ambiguity, also for the affected residues. This combined approach enabled us to achieve sequence-specific assignment for 90% of the backbone resonances of wt LBD (Fig. 1). Figure 1c highlights the remaining non-assigned backbone amides on the structure of GR LBD. We find that these are spread throughout the protein; conversely, residue-specific assignments are available for all regions of the protein, thereby enabling high-resolution studies of ligand and co-regulator peptide binding.

**Fig. 2:**
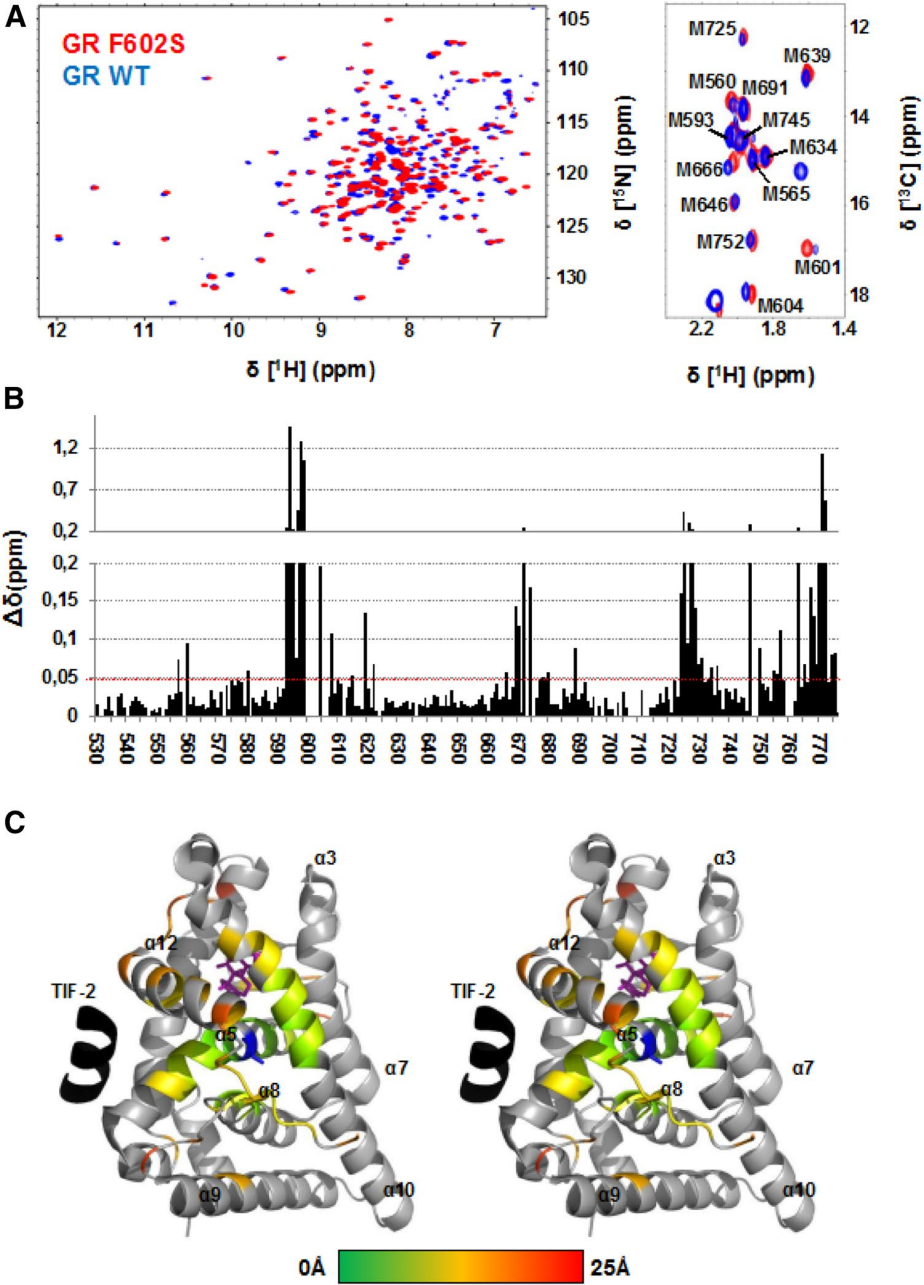
Perturbations of chemical shifts by introducing the stabilizing mutation F602S into GR LBD. **a** Superposition of the ^1^H–^15^N TROSY spectra (left) and the methionine methyl region of the ^1^H–^13^C HSQC spectra of F602S (red) and wildtype (blue) GR LBD T529–K777. **b** Histogram showing the backbone amide chemical shift perturbation caused by the mutation, Δδ = [(Δδ(^15^N))^2^/6.5 + Δδ(^1^H)^2^]^1/2^, plotted versus residue number. The vertical axis is divided into two parts with different scales. **c** Residues with amide chemical shift perturbations Δδ > 0.05 ppm (indicated by the red line in **b**) are highlighted in a color gradient from green to red coding for the distance (between CA atoms) from the site of mutation, PDB id 4UDC (Edman et al. 2015). Dexamethasone is highlighted purple. The co-peptide is highlighted in black .The mutated residue F602S is located in the center of the structure and highlighted in blue

We also carried out residue-specific assignments of methionine methyl groups by systematically introducing residue-specific Met-to-Leu mutations and observing the resulting changes in ^13^C HSQC spectra. This strategy resulted in complete assignments of all methionine methyl groups (Fig. 2a), which will serve as advantageous probes in future studies of conformational dynamics.

Triple resonance spectra of the F602S mutant allowed us to transfer the backbone amide and CA resonance assignment to this commonly used single mutant construct, resulting in approximately 94% completeness for these resonances (Fig. 2). Notably, chemical shift perturbations are observed for residues located far away from the site of mutation (Fig. 2b, c), suggesting that these long-range effects on structure and dynamics might be involved in the increased agonistic effect of the F602S variant.

The assigned backbone ^1^H, ^13^C, and ^15^N, as well as ^13^CεH_3_ methionine chemical shifts of wildtype human GR LBD and of the F602S mutant variant have been deposited in the Biological Magnetic Resonance Bank (BMRB) under accession codes 26756 and 26757, respectively. The assignment statistics are summarized in Table 1.

**Table 1:** Assignment statistics

Resonance	wt GR LBD	F602S GR LBD
N–H	216/240 non-proline residues (90%)	221/240 non-proline residues (92%)
C	219/249 (88%)	–
CA	230/249 (92%)	239/249 (96%)
CεH_3_	13/13 (100%)	13/13 (100%)

This work establishes a solid basis for solution studies of wt GR LBD to monitor conformational and dynamical changes induced by various hormones, synthetic ligands, and co-regulator peptides, as well as more detailed studies on the effect of stabilizing mutations that might lead to increased agonistic effects.

## Electronic supplementary material

Below is the link to the electronic supplementary material.

Supplementary material 1 (PDF 1954 KB)
