# Supplementary material for: Backbone 1H, 13C, and 15N resonance assignments of the ligand binding domain of the human wildtype glucocorticoid receptor and the F602S mutant variant
Source: Biomol NMR Assign. 2018 Apr 17;12(2):263–8. doi: 10.1007/s12104-018-9820-9 (PMC6132842; doi:10.1007/s12104-018-9820-9)
Supplement: Supplementary file 1 — Supplementary material 1 (PDF 1954 KB) [file 12104_2018_9820_MOESM1_ESM.pdf]

Supplementary Information

Backbone ^1H, ^{13}C, and ^{15}N resonance assignments of the ligand binding domain of the human wildtype glucocorticoid receptor and the F602S mutant variant

Christian Köhler[1], Göran Carlström[3], Stefan Tångefjord[2], Tineke Papavoine[1], Matti Lepistö[1], Karl Edman[2]*, Mikael Akke[4]*

[1]Respiratory, Inflammation and Autoimmunity, IMED Biotech Unit, AstraZeneca, Gothenburg, Sweden; [2]Discovery Sciences, IMED Biotech Unit, AstraZeneca, Gothenburg, Sweden; [3]Center for Analysis and Synthesis, and [4]Biophysical Chemistry, Center for Molecular Protein Science, Department of Chemistry, Lund University, Sweden

To whom correspondence should be addressed:
MA, mikael.akke@bpc.lu.se; KE, karl.edman@astrazeneca.com

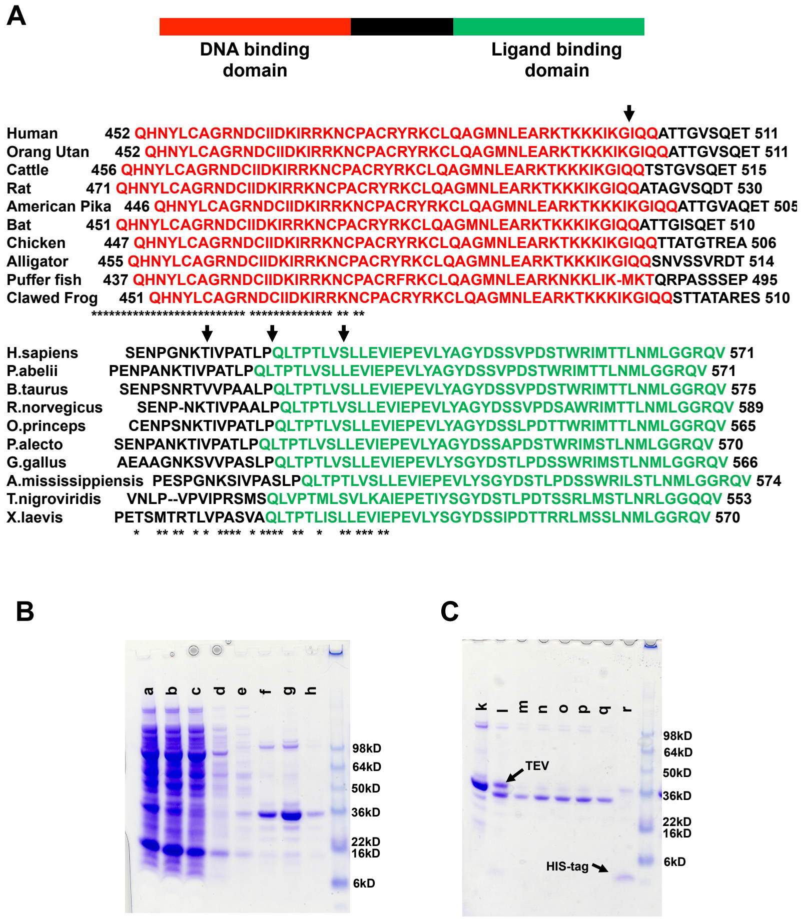

Figure S1: Expression and purification of wildtype GR LBD

A: Sequence alignment of the hinge region between the DNA binding domain (DBD) and ligand binding domain (LBD) of GR orthologs. Arrows mark the starting position of the 4 tested GR LBD fragments: I500–K777, N514–K777, V521–K777 and T529–K777.

B: Purification of His-tagged wt GR LBD monitored by SDS PAGE (4–12%). The different lanes show: a, sonicated cell lysate; b, cleared cell lysate; c, nickel affinity column flow-through; d, nickel affinity column wash; e-h, elution. His-tagged wt GR LBD T529–K777 elutes at 31 kDa.

C: Purification of wt GR LBD monitored by SDS PAGE (4–12%) following His-tag cleavage and a second step of nickel affinity separation. The different lanes show: k, His-tagged wt GR LBD; l, wt GR LBD after His-tag cleavage using TEV protease; m-q, flowthrough containing wt GR LBD; r, elution of TEV protease and His tag

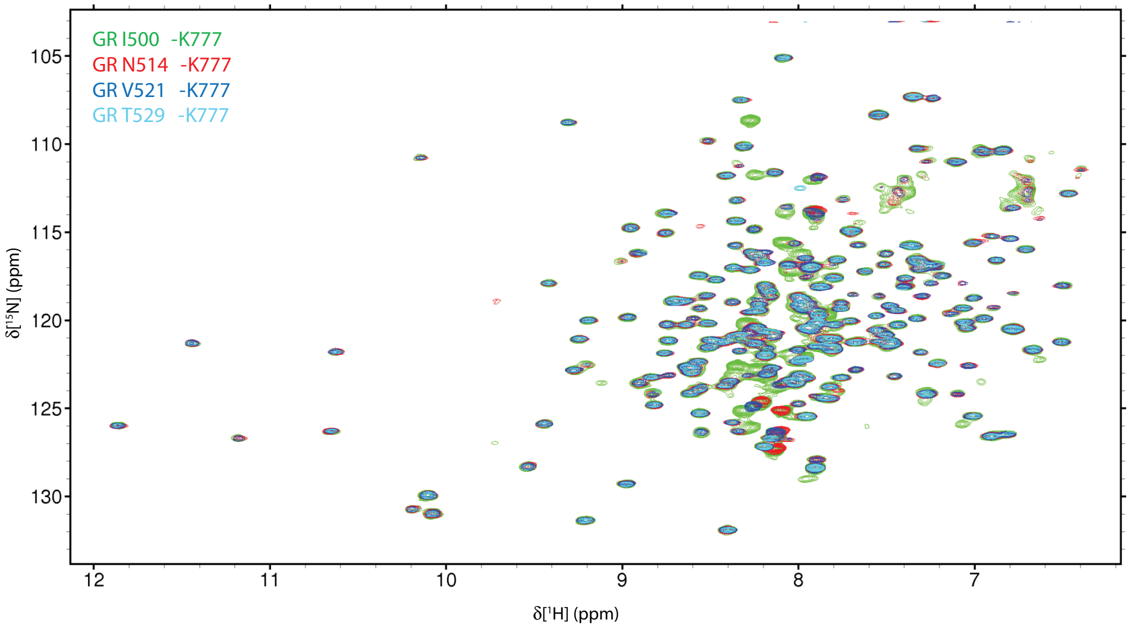

Figure S2. GR LBD T529–K777 retains full structural integrity
Superposition of ^{1}H–^{15}N TROSY NMR spectra of four GR LBD fragments of different lengths. The backbone amide signals of the shortest fragment GR T529–K777 (cyan) superimpose perfectly with signals from the longer fragments, demonstrating that the LBD conformation is identical in all four fragments. The spectrum of GR T529–K777 lacks the intense and overlapped signals in the ^{1}H region around 8 ppm that are characteristic of flexible unstructured protein segments; by contrast, the other fragments show increasing numbers of peaks in this region with increasing length of the N-terminal extensions.
